# Care Partner Views On A Prognosis-Informed Dementia Roadmap

**DOI:** 10.1093/geroni/igaf122.3465

**Published:** 2025-12-31

**Authors:** Nancy Schoenborn, Halima Amjad

**Affiliations:** Johns Hopkins University School of Medicine, Baltimore, Maryland, United States; Johns Hopkins University School of Medicine, Baltimore, Maryland, United States

## Abstract

Almost all older adults with Alzheimer’s disease and related dementias (ADRD) receive help from care partners. An important unmet need in ADRD care partner support is anticipatory guidance and individualized prognostic information. Given a growing compendium of prognostic tools for older adults, including specifically for people living with dementia (PLWD), the science is ripe for developing a prognosis-informed roadmap in PLWD. However, prognostic tools will have little or negative impact if they are not relevant or acceptable to end-users. We aimed to explore care partners’ ADRD prognosis information needs and preferences using qualitative interviews. The study is actively recruiting, with 9 completed interviews thus far. Interviews were analyzed using qualitative thematic analysis. We found that care partners often reported not receiving adequate guidance at the time of ADRD diagnosis. Care partners consider multiple prognostic outcomes important, including life expectancy, likelihood of unsafe behaviors, need for supervision and personal care assistance, and likelihood of mobility challenges. Outcomes that impact living arrangements and safety were the highest priority. Care partners were uniformly accepting of the potential uncertainty inherent in predictions and would nonetheless find the prognostic information useful. Some preferred more personalized predictions while others preferred more general guidance. Care partners reported that the prognosis information would impact healthcare decisions as well as finances, living arrangements, and family members’ life plans. Our preliminary findings demonstrate that ADRD care partners strongly desire prognosis information for anticipatory guidance despite potential uncertainties and prioritize predictions about safety challenges and care needs as the most important.

